# Alcohol Drinking and Bladder Cancer Risk From a Pooled Analysis of Ten Cohort Studies in Japan

**DOI:** 10.2188/jea.JE20190014

**Published:** 2020-07-05

**Authors:** Hiroyuki Masaoka, Keitaro Matsuo, Isao Oze, Hidemi Ito, Mariko Naito, Keiko Wada, Chisato Nagata, Tomio Nakayama, Yuri Kitamura, Atsuko Sadakane, Akiko Tamakoshi, Ichiro Tsuji, Yumi Sugawara, Norie Sawada, Tetsuya Mizoue, Manami Inoue, Keitaro Tanaka, Shoichiro Tsugane, Taichi Shimazu

**Affiliations:** 1 Division of Cancer Epidemiology and Prevention, Aichi Cancer Center Research Institute, Nagoya, Japan; 2 Department of Urology, Graduate School of Medical Sciences, Kyushu University, Fukuoka, Japan; 3 Department of Epidemiology, Nagoya University Graduate School of Medicine, Nagoya, Japan; 4 Division of Cancer Information and Control, Aichi Cancer Center Research Institute, Nagoya, Japan; 5 Department of Oral Epidemiology, Hiroshima University Graduate School of Biomedical and Health Sciences, Hiroshima, Japan; 6 Department of Epidemiology and Preventive Medicine, Gifu University Graduate School of Medicine, Gifu, Japan; 7 Screening Assessment and Management Group, Center for Public Health Sciences, National Cancer Center, Tokyo, Japan; 8 Department of Social Medicine, Osaka University Graduate School of Medicine, Osaka, Japan; 9 Department of Epidemiology, Radiation Effects Research Foundation, Hiroshima, Japan; 10 Department of Public Health, Hokkaido University Graduate School of Medicine, Sapporo, Japan; 11 Division of Epidemiology, Department of Public Health and Forensic Medicine, Tohoku University Graduate School of Medicine, Sendai, Japan; 12 Epidemiology and Prevention Group, Center for Public Health Sciences, National Cancer Center, Tokyo, Japan; 13 Department of Epidemiology and International Health, International Clinical Research Center, National Center for Global Health and Medicine, Tokyo, Japan; 14 Department of Preventive Medicine, Faculty of Medicine, Saga University, Saga, Japan

**Keywords:** alcohol drinking, bladder cancer, cohort study, Japan, pooled analysis

## Abstract

**Background:**

The association of alcohol drinking with bladder cancer risk remains unclear in East Asian populations. Aldehyde dehydrogenase 2 (ALDH2) enzyme oxidizes alcohol-metabolized carcinogenic acetaldehyde into acetate. It is well known that the inactive ALDH2 carriers, specific to East Asian populations, have an increased risk of several cancer types because of increased exposure to acetaldehyde after alcohol consumption. The aim of this study was to examine the association between alcohol drinking and bladder cancer risk using data from ten population-based prospective cohort studies in Japan, where approximately 40% of the population has inactive ALDH2 enzyme.

**Methods:**

We analyzed 340,497 Japanese participants with average follow-up of 13.4 years. The association between alcohol drinking and bladder cancer risk was evaluated using Cox regression models within each study, and random-effects models were used to estimate pooled hazard ratios (HRs) with corresponding 95% confidence intervals (CIs).

**Results:**

During 4,729,071 person-years, 936 men and 325 women were newly diagnosed with bladder cancer. Our results showed no evidence of significant association between alcohol drinking and bladder cancer risk even among men who consumed alcohol of ≥69 g/week, with HR of 1.02 (95% CI, 0.79–1.33). The null result was observed consistently among women.

**Conclusions:**

Our findings do not support an association between alcohol drinking and bladder cancer risk in the Japanese, at least without consideration of the polymorphisms of alcohol-metabolizing enzymes.

## INTRODUCTION

According to the GLOBOCAN 2018 database, bladder cancer is the 10th most common cancer worldwide, responsible for an estimated 549,000 new cases and 200,000 deaths in 2018.^1^ Cigarette smoking is a leading risk factor for bladder cancer, accounting for about 50% of all cases.^2^^,^^3^ Therefore, smoking cessation has been recommended to prevent bladder cancer. Despite efforts to promote smoking cessation, the age-standardized incidence of bladder cancer among Japanese men has been increasing, from 10.6 per 100,000 in 2005 to 14.7 per 100,000 in 2010.^1^ Further primary prevention through identifying a modifiable risk factor other than cigarette smoking and avoiding its exposure is essential to decrease bladder cancer incidence.

Alcohol consumption is recognized as a risk factor for several types of cancer, including oral cavity, head and neck, esophagus, breast, liver, and colorectum cancer.^4^ A previous meta-analysis showed no significant association between alcohol drinking and bladder cancer, although the analysis included only two East Asian studies.^5^ Thus, the impact of alcohol consumption on bladder cancer risk remains unclear in East Asian populations.

Approximately 540 million people in the world, mainly in East Asian countries, have been estimated to carry an inactive aldehyde dehydrogenase 2 (ALDH2) enzyme, which oxidizes alcohol-metabolized acetaldehyde into acetate.^6^ The ALDH2 enzyme activity is dependent on the polymorphism of the *ALDH2* gene (rs671).^6^ Those with inactive *ALDH2* alleles (inactive ALDH2 carriers) have been shown to be exposed to higher concentration of acetaldehyde after alcohol consumption, and alcohol-related acetaldehyde is classified by the International Agency for Research on Cancer (IARC) as a group 1 carcinogen.^7^

Our previous case-control study suggested that those with inactive *ALDH2* alleles showed increased risk of bladder cancer among drinkers.^8^ Furthermore, moderate drinkers with flushing response, most of whom were considered to carry inactive *ALDH2* alleles, had a higher risk of bladder cancer than those without flushing response in the Japanese cohort study.^9^ These two epidemiological studies consistently indicate that drinkers with inactive *ALDH2* alleles may have an increased risk of bladder cancer. Given that the inactive *ALDH2* alleles are specific to East Asian populations, alcohol drinking may have a strong effect of bladder cancer risk in East Asian populations compared with Western populations, implying carcinogenic effects of alcohol-related acetaldehyde on the bladder.

To address this hypothesis, we examined the association of alcohol drinking with bladder cancer from a pooled analysis of ten population-based cohort studies in Japan, where approximately 40% of the population has inactive ALDH2 enzyme, despite some variation across areas.^10^^,^^11^

## METHODS

### Participants

Participants were collected from population-based prospective cohort studies in Japan. Inclusion criteria for this pooled analysis have been described elsewhere.^12^ Ten studies met the criteria: the Japan Public Health Center-based Prospective study (JPHC-I and -II),^13^ the Japan Collaborative Cohort Study (JACC),^14^ the Miyagi Cohort Study (MIYAGI-I),^15^ the Three-Prefecture Cohort Study in Miyagi (MIYAGI-II),^16^ the Three-Prefecture Cohort Study in Aichi (AICHI),^16^ the Three-Prefecture Cohort Study in Osaka (Osaka),^16^ the Takayama Study (TAKAYAMA),^17^ the Ohsaki Cohort Study (OHSAKI)^18^ and the Life-Span study (LSS).^19^ The selected characteristics of each study are shown in eTable 1. Participants with a past history of any cancer at baseline, with missing information on alcohol consumption or smoking, and with exposure to radiation of atomic bomb of equal to or more than 100 mGy (for LSS) were excluded. Data on 340,497 participants were finally analyzed. The study protocols were approved by the institutional review boards of respective study centers.

### Exposure data

Information on alcohol consumption and smoking was collected using self-administered questionnaires in each study at baseline. According to frequency and amount of alcohol consumption, alcohol drinking was categorized as follows: non-drinkers (never and former drinkers), occasional drinkers (<1/week) and regular drinkers (≥1/week: 0.1–22.9, 23.0–45.9, 46.0–68.9, or ≥69 g/day of ethanol) for men. For women, regular drinkers were summarized into one category because a preliminary analysis showed only about 2% of the women consumed more than 23 g/day of alcohol in the JPHC-I and -II (data not shown). Correlation coefficients between self-reported alcohol consumption and diet records were 0.79 in men and 0.44 in women for the JPHC-I,^20^ 0.59 in men and 0.40 in women for the JPHC-II,^21^ 0.77 in men and 0.71 in women for the MIYAGI,^22^ 0.72 in men and 0.64 in women for the TAKAYAMA,^23^ 0.70 in men for the OHSAKI, respectively.^24^ Similar questionnaires were used in the other cohort studies, although validation was not conducted.

### Statistical analysis

Cancer cases were identified through population-based cancer registries and active patient notification from major local hospitals. Cases were coded using the International Classification of Diseases for Oncology, Third Edition (ICD-O-3) or the International Classification of Diseases and Health Related Problems, Tenth Revision (ICD-10).

Person-years of follow-up were counted from the date of baseline survey in each study until the date of bladder cancer diagnosis, migration from the study area, death or the end of follow up, whichever came first. Cox regression models were used to estimate hazard ratios (HR) with corresponding 95% confidence intervals (CI) in the following three models within each study. Model 1 was adjusted for age, area (for JPHC, JACC and LSS), and smoking (pack-year category for men [0, 0.1–20, 20.1–40, or >40], smoking status category for women [never, former, or current]). In model 2, cases diagnosed within 3 years from baseline were excluded. In model 3, the analysis was restricted to never smokers to minimize confounding by smoking. Furthermore, in model 4, the analysis was restricted to ever smokers to examine interaction between smoking and drinking. Trend associations were estimated by calculating the regression coefficient per 10 g of ethanol increase and its standard error among current drinkers. Random-effects models were used to calculate pooled estimates. Heterogeneity among studies was assessed using Q-statistics and I^2^ statistics. Statistical analyses were conducted using STATA statistical software version 13.1 (StataCorp LP, College Station, TX, USA). All two-sided *P* values <0.05 were considered statistically significant.

## RESULTS

During the average follow-up of 13.4 years (4,729,071 person-years), 936 men and 325 women were diagnosed with bladder cancer. The frequencies of regular drinking were 71% among men and 14% among women. When adjusted for age, area and smoking (model 1), no evidence of increased bladder cancer risk was observed for regular drinking (Table 1). Even among men who consumed alcohol of ≥69 g/week, HR was 1.02 (95% CI, 0.79–1.33). No significant linear trend between bladder cancer risk and alcohol consumption was also seen (HR 1.01; 95% CI, 0.99–1.03). These results were consistent with the analysis restricted to never smokers and ever smokers (model 3 and model 4), suggesting no significant interaction between smoking and drinking regarding bladder cancer risk. Furthermore, for women, regular drinkers showed no increased risk of bladder cancer compared with non-drinkers, as shown in Table 2.

**Table 1.** HRs and 95% CIs of bladder cancer risk according to drinking status and amount of alcohol drinking among current drinkers in men

	Drinking status and amount of alcohol drinking among regular drinkers (g/week of ethanol)							Heterogeneity									
	
	Non-drinker	Occasional drinker (<1/week)	Regular drinker (≥1/week)				Unit HR (per 10 g of ethanol among current drinkers)	<1/week		0.1–22.9		23.0–45.9		46.0–68.9		≥69.0	
					
			0.1–22.9	23.0–45.9	46.0–68.9	≥69.0		*P*	I^2^, %	*P*	I^2^, %	*P*	I^2^, %	*P*	I^2^, %	*P*	I^2^, %
Number of subjects	37,719	18,856	28,653	30,985	24,214	16,868											
Person-years	486,764	249,267	389,952	435,337	342,435	241,327											
Number of cases	247	80	156	199	160	94											
HR1 (model 1)^a^	1.00 (Ref)	0.89 (0.68–1.17)	0.95 (0.77–1.17)	1.07 (0.88–1.30)	1.13 (0.91–1.39)^i^	1.02 (0.79–1.33)^i^	1.01 (0.99–1.03)	0.793	0	0.479	0	0.109	38	0.528	0	0.743	0
HR2 (model 2)^b^	1.00 (Ref)	0.90 (0.66–1.24)	0.93 (0.74–1.17)^f^	1.12 (0.90–1.38)	1.11 (0.88–1.41)^i^	1.02 (0.76–1.36)^k^	1.01 (0.99–1.03)	0.860	0	0.589	0	0.246	21	0.275	19	0.857	0
HR3 (model 3)^c^	1.00 (Ref)	1.08 (0.53–2.21)^e^	0.93 (0.55–1.59)^g^	1.29 (0.77–2.16)^h^	0.71 (0.32–1.57)^j^	1.50 (0.62–3.66)^l^	1.00 (0.94–1.06)	0.973	0	0.995	0	0.842	0	0.952	0	0.768	0
HR4 (model 4)^d^	1.00 (Ref)	0.91 (0.67–1.23)	0.95 (0.75–1.19)	1.05 (0.85–1.30)	1.16 (0.94–1.45)^i^	0.99 (0.75–1.30)^i^	1.02 (0.99–1.04)	0.574	0	0.301	16	0.142	33	0.571	0	0.690	0

CI, confidence interval; HR, hazard ratio.

^a^Model 1: adjusted for age (continuous), public health center area (JPHC, JACC and LSS only) and smoking (pack-year category; 0, 0–20, 20–40, >40).

^b^Model 2: model 1 excluding early cases (within the first 3 years).

^c^Model 3: model 1 restricted to never smokers.

^d^Model 4: model 1 restricted to ever smokers.

^e^HRs are pooled from JPHC I/II, MIYAGI I/II, OSAKA and TAKAYAMA.

^f^HRs are pooled from JPHC I/II, JACC, MIYAGI I/II, TAKAYAMA, OHSAKI and LSS.

^g^HRs are pooled from JPHC I/II, JACC, MIYAGI I/II, TAKAYAMA and OHSAKI.

^h^HRs are pooled from JPHC I/II, JACC, MIYAGI I/II, AICHI, TAKAYAMA and OHSAKI.

^i^HRs are pooled from JPHC I/II, JACC, MIYAGI I, AICHI, OSAKA, TAKAYAMA, OHSAKI and LSS.

^j^HRs are pooled from JPHC I/II, JACC, MIYAGI I, TAKAYAMA and OHSAKI.

^k^HRs are pooled from JPHC I/II, JACC, MIYAGI I, OSAKA, TAKAYAMA, OHSAKI and LSS.

^l^HRs are pooled from JPHC I/II, JACC and TAKAYAMA.

**Table 2.** HRs and 95% CIs of bladder cancer risk according to drinking status in women

	Drinking status			Heterogeneity			
	
	Non-drinker	Occasional drinker (<1/week)	Regular drinker (≥1/week)	<1/week		≥1/week	
	
				*P*	I^2^, %	*P*	I^2^, %
Number of subjects	130,609	26,498	26,095				
Person-years	1,854,786	364,524	364,679				
Number of cases	246	41	38				
CR (per 100,000)	13.3	11.2	10.4				
HR1 (model 1)^a^	1.00 (Ref)	1.31 (0.91–1.88)	0.96 (0.66–1.40)^d^	0.810	0	0.909	0
HR2 (model 2)^b^	1.00 (Ref)	1.51 (1.02–2.24)	1.10 (0.74–1.62)^e^	0.563	0	0.901	0
HR3 (model 3)^c^	1.00 (Ref)	1.48 (1.00–2.19)	1.11 (0.70–1.75)^f^	0.700	0	0.651	0

CI, confidence interval; HR, hazard ratio.

^a^Model 1: adjusted for age (continuous), public health center area (JPHC, JACC and LSS only) and smoking (status category; never, former, current).

^b^Model 2: model 1 excluding early cases (within the first 3 years).

^c^Model 3: model 1 restricted to never smokers.

^d^HRs are pooled from JPHC I/II, JACC, MIYAGI I, AICHI, OSAKA, TAKAYAMA, OHSAKI and LSS.

^e^HRs are pooled from JPHC I/II, JACC, MIYAGI I, OSAKA, TAKAYAMA, OHSAKI and LSS.

^f^HRs are pooled from JPHC I/II, JACC, OSAKA, TAKAYAMA and LSS.

## DISCUSSION

Our large-scale pooled analysis showed alcohol consumption was not associated with bladder cancer risk among Japanese populations. This finding was consistent with a previous meta-analysis, mainly consisting of Western population studies.^5^

A mechanistic role of alcohol drinking in the development of bladder cancer is plausible, given carcinogenic effects of alcohol-derived acetaldehyde. Acetaldehyde forms protein and DNA with adducts, leading to genotoxic and mutagenic effects.^25^ Carcinogenic effects of alcohol-derived acetaldehyde have been also shown in epidemiological studies of esophagus, head and neck, and gastric cancer.^26^ Alcohol consumption caused increased concentration of urinary acetaldehyde among alcohol drinkers,^27^ suggesting increased carcinogenic effects on the bladder among drinkers.

On the contrary, the association between alcohol consumption and bladder cancer risk was not observed in the present study, despite the high prevalence of inactive *ALDH2* alleles in Japan. This may be explained by our previous finding that those with active *ALDH2* alleles showed no increased risk of bladder cancer, even though they drank heavily.^8^ Moderate to heavy drinkers have more frequently active *ALDH2* alleles than never to light drinkers.^8^ Thus, the frequent active *ALDH2* alleles of moderate to heavy drinkers may lead to no significant association between alcohol consumption and bladder cancer. In addition, those with inactive *ALDH2* alleles were shown to consume less amount of alcohol because of acetaldehyde-related adverse effects, such as flushing, palpitations, nausea, and headache.^28^ The carcinogenetic effect of the inactive *ALDH2* alleles on the bladder may be attenuated by decreased amount of alcohol consumption in inactive ALDH2 carriers. Another explanation for the null association was the involvement of alcohol dehydrogenase 1B (ADH1B). The ADH1B enzyme metabolizes alcohol to acetaldehyde. Our previous study suggested that the fast alcohol-metabolizing *ADH1B* alleles, more prevalent in East Asian countries than Western countries, had a decreased risk of bladder cancer possibly due to reduced amount of alcohol consumption.^8^ This protective effect of fast alcohol-metabolizing *ADH1B* alleles may play some role in the null association. Because the present analysis did not examine either *ALDH2* or *ADH1B* polymorphisms, these explanations remain just hypotheses. Nevertheless, the analysis without consideration of the genetic polymorphisms of alcohol-metabolizing enzymes may obscure the true association between alcohol consumption and bladder cancer given the carcinogenetic effects of these polymorphisms. The present null result may, therefore, suggest the need for future studies that investigate bladder cancer risk associated with alcohol consumption stratified by the *ALDH2* and *ADH1B* polymorphisms in East Asians.

Several limitations should be noted. First, occupational information was unavailable, although occupational chemical exposure was an established risk factor for bladder cancer.^3^ Second, the limited number of women drinkers did not allow for investigation of the dose-response relationship for alcohol consumption in women. Despite these limitations, the data of this study were derived from large-scale population-based cohort studies in Japan, where those with inactive *ALDH2* alleles are frequent unlike Western countries. The analysis restricted to never smokers allowed our results to remove confounding by smoking. Furthermore, prospective data collection can reduce information bias inherent in retrospective study designs.

In conclusion, our results do not support an association between alcohol consumption and bladder cancer risk in the Japanese, without consideration of polymorphisms in alcohol-metabolizing enzymes.

## APPENDIX A. SUPPLEMENTARY DATA

eTable 1. Characteristics of the cohort studies in the present pooled analysis

## APPENDIX B.

Research group members are listed at the following site (as of August 2018): http://epi.ncc.go.jp/en/can_prev/796/7955.html
