# Supplementary material for: Alcohol Drinking and Bladder Cancer Risk From a Pooled Analysis of Ten Cohort Studies in Japan
Source: J Epidemiol. 2020 Jul 5;30(7):309–13. doi: 10.2188/jea.JE20190014 (PMC7280052; doi:10.2188/jea.JE20190014)
Supplement: Supplementary file 1 [file je-30-309-s001.pdf]

eTable 1. Characteristics of the cohort studies in the present pooled analysis

Study	Population	Age range at baseline, y	Year of baseline survey	Population size	Response rate of the baseline questionnaire	Method of follow-up	For the present pooled analysis							
							Age range, y	Last follow-up year	Mean follow-up period, y	Outcome	Size of the cohort		Number of bladder cancer cases	
											Men	Women	Men	Women
JPHC-I	Japanese residents of 5 public health center areas in Japan	40–59	1990	61,595	82%	Cancer registry and death certificate	40–59	2012	20.1	Incidence	19,778	21,606	147	42
JPHC-II	Japanese residents of 6 public health center areas in Japan	40–69	1993– 1994	78,825	80%	Cancer registry and death certificate	40–69	2012	16.8	Incidence	24,606	28,213	196	66
JACC	Residents from 45 areas throughout Japan	40–79	1988–1990	110,585	83%	Cancer registry (selected areas: 22) and death certificate	40–79	2009	13.4	Incidence	20,426	32,889	116	52
MIYAGI-I	Residents of 14 municipalities in Miyagi Prefecture, Japan	40–64	1990	47,605	92%	Cancer registry and death certificate	40–64	2007	16.2	Incidence	18,648	16,456	109	26

MIYAGI-II	Residents of 3 municipalities in Miyagi Prefecture, Japan	40+	1984	31,345	94%	Cancer registry and death certificate	40+	1992	7.6	Incidence	9,398	10,730	26	5
AICHI	Residents of 2 municipalities in Aichi Prefecture, Japan	40–103	1985	33,529	90%	Cancer registry and death certificate	40–103	2000	11.5	Incidence	14,996	14,226	58	22
OSAKA	Residents of 4 municipalities in Osaka Prefecture, Japan	40–97	1983–1985	35,755	85%	Cancer registry and death certificate	40–97	2000	12.3	Incidence	11,304	15,936	39	17
TAKAYAMA	Residents of Takayama city, Gifu Prefecture, Japan	35+	1992	31,552	85%	Cancer registry and death certificate	35–101	2008	13.6	Incidence	13,824	14,831	116	33
OHSAKI	Residents of 14 municipalities in Miyagi Prefecture, Japan	40–79	1994	52,029	95%	Cancer registry and death certificate	40–79	2005	9.3	Incidence	18,439	17,528	85	40

LSS	Atomic bomb survivors in Hiroshima and Nagasaki	0–91	1950	120,321	100%	Cancer registry and death certificate	46–104	2003	10.8	Incidence	5,876	10,787	44	22
Total				603,141							157,295	183,202	936	325

AICHI, Aichi Cohort Study; JACC, The Japan Collaborative Cohort Study; JPHC, Japan Public Health Center-based prospective Study; LSS, Life-Span Study; MIYAGI, The Miyagi Cohort Study; OHSAKI, Ohsaki Cohort Study; OSAKA, Osaka Cohort Study; TAKAYAMA, Takayama Study.
